# Case report: Fatal *Legionella* infection diagnosed via by metagenomic next-generation sequencing in a patient with chronic myeloid leukemia

**DOI:** 10.3389/fmed.2023.1266895

**Published:** 2023-11-22

**Authors:** Chunhong Bu, Shuai Lei, Linguang Chen, Yanqiu Xie, Guoli Zheng, Liwei Hua

**Affiliations:** ^1^Department of Intensive Care Unit, Affiliated Hospital of Chengde Medical University, Chengde, China; ^2^Department of Pharmacy, Affiliated Hospital of Chengde Medical University, Chengde, China; ^3^Department of Hepatobiliary Surgery, Affiliated Hospital of Chengde Medical University, Chengde, China; ^4^Department of Radiology, Affiliated Hospital of Chengde Medical University, Chengde, China

**Keywords:** myeloid leukemia, *Legionella* pneumonia, metagenomic next-generation sequencing, bloodstream infection, case report

## Abstract

*Legionella* is an aerobic, gram-negative, intracellular pathogen and is an important cause of community-acquired pneumonia. *Legionella pneumophila* is the most common causative agent of *Legionella* pneumonia. Clinical diagnosis of *Legionella* pneumonia is challenging due to the lack of specific clinical manifestations and the low positive rates of conventional pathogen detection methods. In this study, we report a case of a patient with chronic myeloid leukemia who developed rigors and high fever after chemotherapy and immunotherapy. Chest computed tomography revealed consolidation in the left lower lobe of the lung and ground-glass opacities in both lower lobes. Multiple blood cultures showed *Escherichia coli*, *Staphylococcus aureus*, *Bacillus licheniformis*, and positive results in the β-D-glucan test (G test). The patient was treated with various sensitive antimicrobial agents, including meropenem plus fluconazole, meropenem plus carpofungin, and vancomycin. Unfortunately, the patient’s condition gradually worsened and eventually resulted in death. On the following day of death, metagenomic next-generation sequencing (mNGS) of 1whole blood revealed *L. pneumophila* pneumonia with concurrent bloodstream infection (blood mNGS reads 114,302). These findings suggest that when conventional empirical antimicrobial therapy proves ineffective for critically ill patients with pneumonia, the possibility of combined *Legionella* infection must be considered, and mNGS can provide a diagnostic tool in such cases.

## 1 Introduction

*Legionella* is an aerobic, gram-negative rod bacterium widely found in warm and humid natural environments. *Legionella* pneumonia (LP) is a lung infection caused by *Legionella* species and can involve extrapulmonary organs. Although LP currently accounts for only 2–9% of community-acquired pneumonia (CAP) cases (1), its incidence is increasing, and the disease can develop into severe pneumonia. At present, there is insufficient large-sample epidemiological data on LP in China. Meanwhile, data from the European Union/European Economic Area (EU/EEA) has shown an upward trend in the notification rate for *Legionella* disease. This rate increased from 1.2 to 1.4/100,000 population between 2012 and 2016, and further increased to 1.8–2.2 during 2017–2019 (2). Given the limited awareness about the disease among clinicians and the difficulty of early diagnosis, the actual incidence of LP is likely higher. LP is more common in immunocompromised individuals, with hematologic malignancies being one of the high-risk factors (3). A case of leukemia-associated LP diagnosed via metagenomic next-generation sequencing (mNGS) was previously reported in the literature (4). However, the patient had no accompanying bloodstream infection, and the mNGS sequence count was low. In this paper, we report the case of a patient with chronic myeloid leukemia who developed fever and pneumonia after chemotherapy and immunotherapy. Multiple “pathogenic bacteria” were identified in blood culture, and various “sensitive drugs” were administered. However, the true pathogen, *Legionella*, was overlooked until the patient was diagnosed with LP and bloodstream infection through mNGS (*Legionella pneumophila* sequence count: 114,302; relative abundance: 95.42%). Ineffective treatment before LP diagnosis eventually led to the patient’s death, highlighting the need to consider combined *Legionella* infection in the future.

## 2 Case description

**Figure 1:**
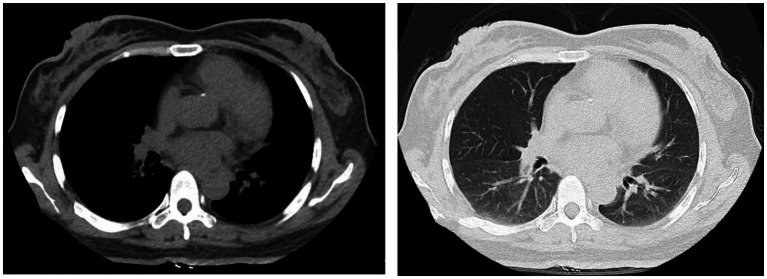
Chest CT performed on June 13, 2022, showed no definite abnormalities.

The patient, a 49-year-old female, was admitted to the hospital on June 11, 2022, for the third cycle of chemotherapy due to a diagnosis of chronic myeloid leukemia that had persisted for over 3 months. She had a medical history of hypertension and type 2 diabetes. On admission, her vital signs were as follows: temperature 36°C, blood pressure 96/68 mmHg, heart rate 84 beats per minute, and respiratory rate 18 breaths per minute. Chest computed tomography (CT) performed on June 13 showed no abnormalities (Figure 1). The patient received targeted therapy with imatinib mesylate and chemotherapy with idarubicin combined with cytarabine on June 14. On June 28, the patient developed rigors and fever, with a maximum temperature of 40.4°C.

Blood tests revealed leukopenia with a white blood cell count of 0.1 × 10^^9^/L (normal range: 3.5–9.5 × 10^^9^/L), neutropenia with an absolute neutrophil count of 0 (normal range: 1.8–6.3 × 10^^9^/L), anemia with a hemoglobin level of 78 g/L (normal range: 115–150 g/L), and thrombocytopenia with a platelet count of 5 × 10^^9^/L (normal range: 125–350 × 10^^9^/L). Lymphocyte subset analysis showed an absolute CD3 + CD4+ T lymphocyte count of 195 cells/μL (normal range: 550–1,440 cells/μL) and an absolute CD3 + CD8+ T lymphocyte count of 273 cells/μL (normal range: 320–1,250 cells/μL). The β-D-glucan test (G test) yielded a result greater than 600 pg./mL (normal range: 0–70 pg./mL).

**Figure 2:**
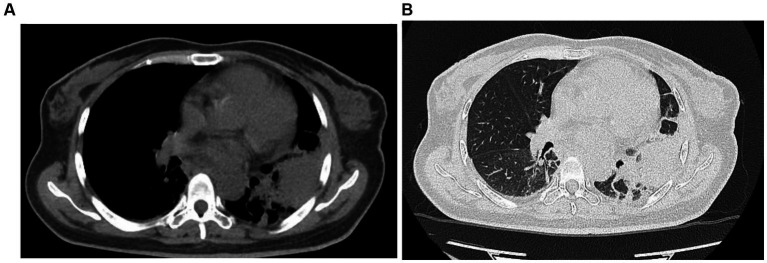
Chest CT performed on July 5, 2022, revealed a large consolidative opacity in the left lower lobe and ground-glass opacities in both lower lobes.

The patient was initially treated with antimicrobial therapy with intravenous meropenem (1.0 g every 8 h) and oral fluconazole (200 mg once daily), which was later switched to intravenous caspofungin (50 mg once daily). However, the symptoms persisted, and the patient developed a cough with productive sputum. On auscultation, decreased breath sounds and crackles were observed in the left lower lung. A follow-up chest CT on July 5 revealed consolidations in the left lower lobe and ground-glass opacities in both lower lobes (Figure 2). Multiple blood cultures tested positive for *Escherichia coli*, *Staphylococcus aureus*, and *Bacillus licheniformis*. Consequently, intravenous vancomycin (1 g every 12 h) was added to the treatment regimen, but the patient’s condition did not improve.

On July 9, the patient was transferred to the ICU due to respiratory and circulatory failure and was subsequently intubated and mechanically ventilated. High-dose vasopressors were administered to maintain circulation. Sputum, tracheal aspirate, and bronchoalveolar lavage fluid cultures yielded negative results, as did *L. pneumophila* IgM. On July 12, whole blood mNGS was performed using the PMSEQ-4500 platform (BGI Co., Ltd., Shenzhen, China). The results, available on July 14, indicated *L. pneumophila* with a sequence count of 114,302 and a relative abundance of 95.42%, with no co-infections detected from other bacteria, fungi, viruses, or suspected background microorganisms. Combined with her medical history, clinical symptoms, physical signs, auxiliary examinations, and mNGS, we eventually diagnosed the patient with *Legionella* pneumonia and bloodstream infection. Unfortunately, the patient succumbed to multiple organ failure (MOF) on July 13. Written informed consent was obtained from the individual for the publication of any potentially identifiable images or data included in this article.

## 3 Discussion

Human *Legionella* infection is primarily caused by *L. pneumophila*. It mainly affects the lower respiratory tract and is more common in the summer and early autumn. A study from Europe showed that 70.7% of *Legionella* infections were community-acquired, 19.9% were travel-related, and 7.3% were healthcare-associated (5). The mortality rate of hospital-acquired LP can reach 50%, significantly higher than that of community-acquired LP (10%) (6). Lupia et al. (7) found that nearly one-quarter of the LP cases occurred in immunocompromised individuals with conditions such as AIDS, cancer, or hematological disorders, and the disease was more likely to progress to severe pneumonia in these patients.

The overall mortality rate of LP is 4–18%, and approximately 20–27% of patients require ICU admission (8). However, in immunocompromised patients who do not receive appropriate antimicrobial treatment in the early stages, the mortality rate can be as high as 80% (9). *Legionella* bacteria are characterized by their inability to grow on routine cultures, atypical clinical presentations, resistance to commonly used beta-lactam antibiotics, and concurrent infections with other bacteria or fungi, making rapid and timely diagnosis difficult. This often leads to the selection of incorrect treatment regimens, thereby missing the optimal treatment window for LP. A study from Korea showed that low platelet count (≤150,000/mm^3^) and delayed antibiotic treatment (>1 day) were independent risk factors for the progression of non-severe LP to severe pneumonia (10). Therefore, high mortality rates are closely related to delayed diagnosis, inappropriate antibiotic selection, and concurrent infections with other pathogens. The present case was a patient with chronic myeloid leukemia who had myelosuppression after chemotherapy. Her condition was complicated by granulocytopenia and severe immunosuppression, which made her susceptible to various infections, including *Legionella*. Although multiple blood cultures showed the growth of various bacteria, the presence of *Legionella* was overlooked; therefore, targeted antimicrobial treatment was not administered.

The clinical manifestations of Legionnaires’ disease are associated with bacterial load, virulence factors, and the patient’s immune status (11). In this case, the patient had underlying chronic leukemia and had undergone multiple chemotherapy and targeted therapy sessions, resulting in immunosuppression and an increased susceptibility to severe infections. Notably, the clinical manifestation of LP is similar to that of other pneumonias and lacks specificity. Common symptoms include fever, respiratory symptoms (such as cough and dyspnea), gastrointestinal symptoms, and neurological symptoms. In some cases, extrapulmonary manifestations such as splenomegaly, splenic rupture, pericarditis, and arthritis may occur (12). Common complications of LP include pleural effusion, acute kidney injury, and rhabdomyolysis (7). The main clinical manifestations in this patient were high fever, cough, and sputum production. Multiple cultures of sputum, tracheal aspirate, and bronchoalveolar lavage fluid were all negative. However, we did not consider atypical pathogens such as *Legionella*, and further targeted investigations were performed to determine the diagnosis.

Early diagnosis followed by timely and appropriate antimicrobial treatment are key factors in reducing the mortality rate of LP. Although bacterial culture currently remains the “gold standard” for LP diagnosis, its strict requirements pose challenges for early diagnosis (13), with only 5% of patients being diagnosed through this approach (12). However, urinary antigen testing is a rapid method for detecting *Legionella* and is considered a frontline diagnostic tool for LP, accounting for 70–80% of confirmed cases in Europe (14). However, the sensitivity of urinary antigen testing for LP caused by *L. pneumophila* serogroup 1 is at most 80–90%; this sensitivity further drops to <50% for LP caused by other *Legionella* species (15). Therefore, the risk of missed diagnoses is high when this approach is used alone.

Conversely, mNGS is a novel and promising approach which combines high-throughput sequencing with bioinformatics analysis and can directly identify pathogen sequences in clinical samples. It has been applied in clinical infectious disease diagnostics, including pneumonia (16). mNGS typically involves the following steps: sample collection, sample processing, and nucleic acid extraction, library construction, high-throughput sequencing, bioinformatics analysis, and interpretation of mNGS results. Numerous studies have demonstrated the increasing advantages of mNGS in pathogen diagnosis. These advantages include rapid diagnosis (approximately 30 h), precision, the ability to detect a wide range of pathogens, and applicability to various types of samples. Additionally, the results are less influenced by previous antibiotics (4, 17–19). However, its clinical application is limited by the high cost, complex laboratory procedures, and susceptibility to contamination. The patient presented with a severe infection and repeatedly tested positive for blood cultures. Despite receiving various antibiotics, four blood cultures and a bronchoalveolar lavage fluid culture were all negative. However, fever remained high and there was no improvement in infection symptoms. The true pathogen could not be identified. Eventually, whole-blood mNGS revealed a high sequence of *L. pneumophila*, with no co-infections detected from other bacteria, fungi, viruses, or suspected background microorganisms. Combined with the patient’s immunosuppressive status, clinical features, imaging and laboratory tests, and mNGS results, the final diagnosis was *Legionella* pneumonia accompanied by bloodstream infection. Due to the patient being clinically dead when the mNGS results were reported, no further examination was conducted. Due to inadequate awareness of LP and general satisfaction with the positive results from multiple blood cultures, the authors failed to perform early mNGS for a definitive diagnosis, resulting in a delay in effective treatment and, ultimately, the patient’s death. In summary, whole blood mNGS is of great significance in the diagnosis of this patient.

Regarding treatment, as *Legionella* is an intracellular bacterium, effective antimicrobial therapy requires selecting antibiotics that can achieve high intracellular drug concentrations and penetrate lung tissue. The 2007 guidelines on CAP management in adults, jointly issued by the Infectious Diseases Society of America and the American Thoracic Society, recommend quinolones and macrolides as first-line agents, with doxycycline as an alternative. For severe *Legionella* infections, the guidelines suggest using a combination of fluoroquinolones and macrolides or levofloxacin (20). The dosage and route of administration should be determined based on disease severity, potential risk factors, patient level of consciousness, and gastrointestinal condition (21). Regarding the duration of treatment, it is recommended to treat immunocompetent patients for a minimum of 2 weeks, while immunosuppressed patients should receive at least 3 weeks of treatment to reduce the risk of relapse after discontinuation (22). Effective treatment relies on appropriate antimicrobial therapy after early diagnosis, proper management of complications, and effective control of potential comorbidities and risk factors.

## 4 Conclusion

LP can be easily overlooked in clinical practice due to the lack of specific clinical manifestations. This study reported a case of secondary LP in a patient with chronic leukemia who died due to delayed diagnosis and ineffective treatment. This highlights the importance of considering the possibility of *Legionella* infection in critically ill patients with pneumonia, especially those who are immunocompromised and remain unresponsive to corresponding antimicrobial therapy, even if definitive microbiological results have been obtained. For such patients, it is crucial to perform *Legionella* testing promptly and include empiric antibiotic therapy targeting *Legionella* upon positive results. If necessary, mNGS can be employed as an early diagnostic approach, followed by targeted treatment to reduce the mortality rate effectively.

## Data availability statement

The original contributions presented in the study are included in the article/supplementary material, further inquiries can be directed to the corresponding author.

## Ethics statement

Written informed consent was obtained from the individual’s next of kin for the publication of any potentially identifiable images or data included in this article.

## Author contributions

CB: Data curation, Writing – original draft. SL: Formal analysis, Writing – original draft. LC: Methodology, Supervision, Writing – original draft. YX: Data curation, Writing – original draft. GZ: Resources, Writing – review & editing. LH: Conceptualization, Resources, Visualization, Writing – review & editing.
